# BDNF Genotype Interacts with Motor Function to Influence Rehabilitation Responsiveness Poststroke

**DOI:** 10.3389/fneur.2016.00069

**Published:** 2016-05-17

**Authors:** Christine T. Shiner, Kerrie D. Pierce, Angelica G. Thompson-Butel, Terry Trinh, Peter R. Schofield, Penelope A. McNulty

**Affiliations:** ^1^Neuroscience Research Australia, Sydney, NSW, Australia; ^2^School of Medical Sciences, University of New South Wales, Sydney, NSW, Australia

**Keywords:** motor rehabilitation, upper limb, stroke genetics, apolipoprotein E, brain-derived neurotrophic factor

## Abstract

**Background:**

Persistent motor impairment is common but highly heterogeneous poststroke. Genetic polymorphisms, including those identified on the brain-derived neurotrophic factor (BDNF) and apolipoprotein E (APOE) genes, may contribute to this variability by limiting the capacity for use-dependent neuroplasticity, and hence rehabilitation responsiveness.

**Objective:**

To determine whether BDNF and APOE genotypes influence motor improvement facilitated by poststroke upper-limb rehabilitation.

**Methods:**

BDNF-Val66Met and APOE isoform genotypes were determined using leukocyte DNA for 55 community-dwelling patients 2–123 months poststroke. All patients completed a dose-matched upper-limb rehabilitation program of either Wii-based Movement Therapy or Constraint-induced Movement Therapy. Upper-limb motor function was assessed pre- and post-therapy using a suite of functional measures.

**Results:**

Motor function improved for all patients post-therapy, with no difference between therapy groups. In the pooled data, there was no significant effect of BDNF or APOE genotype on motor function at baseline, or following the intervention. However, a significant interaction between the level of residual motor function and BDNF genotype was identified (*p* = 0.029), whereby post-therapy improvement was significantly less for Met allele carriers with moderate and high, but not low motor function. There was no significant association between APOE genotype and therapy outcomes.

**Conclusion:**

This study identified a novel interaction between the BDNF-Val66Met polymorphism, motor-function status, and the magnitude of improvement with rehabilitation in chronic stroke. This polymorphism does not preclude, but may reduce, the magnitude of motor improvement with therapy, particularly for patients with higher, but not lower residual motor function. BDNF genotype should be considered in the design and interpretation of clinical trials.

## Introduction

Motor impairment is a common, disabling, and inherently heterogeneous outcome of stroke (1, 2). Patients typically present across a broad clinical continuum and undergo variable and often incomplete recovery of motor function over time and in response to targeted rehabilitation (3). Predicting poststroke prognosis and recovery potential has gained a prominent research focus, with the most common predictive factors being measures of lesion size (4, 5), location (6, 7), corticospinal tract integrity (8, 9), and initial impairment severity (10, 11). While more extensive corticospinal tract damage and more severe baseline impairment are generally associated with poorer prognosis poststroke (10–12), these factors alone cannot fully explain the degree of variability in poststroke motor outcomes and patients’ response to motor therapies (3, 13). In order to optimize rehabilitation and thus maximize poststroke recovery, a deeper understanding of the factors that mediate this residual variability is necessary.

Genetic variation may account for some of the unexplained variance in stroke recovery. In particular, single-nucleotide polymorphisms (SNPs) in genes related to cortical plasticity and neural repair could influence an individual’s capacity for use-dependent plasticity, and hence their responsiveness to poststroke rehabilitation [for review, see Ref. (14)]. Numerous genes of interest continue to emerge in the growing field of stroke genetics (14, 15). Here, we have adopted a candidate gene approach based on two genetic factors with the strongest evidence in subacute stroke and extended this investigation into the chronic setting. The candidate genes are the brain-derived neurotrophic factor (BDNF) and apolipoprotein E (APOE) genes (16).

The BDNF gene encodes for the neurotrophin most abundantly expressed in the brain and involved in neuronal differentiation, survival, and synaptic plasticity (17–19). Approximately 30% of the Caucasian population and a higher percentage of the Asian population possess an SNP (rs6265) in the BDNF gene, resulting in a valine to methionine substitution at codon 66, the Val66Met polymorphism (20). This polymorphism alters the intracellular trafficking and activity-dependent release of BDNF (21, 22), and in healthy cohorts has been associated with a reduced capacity for use-dependent plasticity in the motor cortex (23–26) and impaired motor learning (26).

The BDNF-66Met allele may be detrimental to recovery following stroke (14), but the evidence to date remains contentious (27). Studies have primarily focused on subacute outcomes following spontaneous recovery, where both a significant negative association between Val66Met and stroke outcome (28–31) and a modest or negligible effect have been reported (16, 32–34). There is scant evidence of whether the Val66Met polymorphism influences long-term stroke recovery or responsiveness to targeted therapies. There is some suggestion that it may alter patient responsiveness to non-invasive brain stimulation (35, 36), but to date, no significant effect of this genotype on motor therapy has been identified (13).

Single-nucleotide polymorphisms within the APOE gene are less prevalent but potentially stronger genetic mediators of poststroke recovery (16). This gene encodes for a glycoprotein primarily involved in lipid transport and metabolism, but it also plays an important role in neuronal repair and synaptic remodeling (37, 38). Two SNPs (rs429358 and rs7412) in the APOE gene give rise to three distinct APOE isoforms, ε2 (Cys^112^/Arg^158^Cys), ε3 (Cys^112^/Arg^158^), and ε4 (Cys^112^Arg/Arg^158^) (39). The ε4 isoform is present in only 10–20% of the population (40) but has been strongly implicated in the risk for Alzheimer’s disease (41, 42) and cardiovascular pathology (40, 43). Studies of APOE ε4 in stroke have mainly focused on stroke incidence rather than outcome (43), although emerging evidence suggests that the ε4 allele may have a detrimental effect on poststroke recovery (16, 44–46). Like BDNF, it remains uncertain whether APOE genotype can influence motor function and rehabilitation outcomes more chronically poststroke (13).

Here, we investigated whether BDNF and APOE genotype influence how stroke patients with stable motor function respond to a targeted protocol of upper-limb motor therapy poststroke. Data were collected from a pooled cohort of patients who received a dose-matched protocol of either Constraint-induced Movement Therapy, the current gold standard in upper-limb stroke rehabilitation, or Wii-based Movement Therapy, recently shown to be an engaging and equally efficacious therapy alternative (47). Given that no differences were demonstrated in any measure of upper-limb motor function between these two therapies (47), we did not expect to see differences according to therapy type, but rather according to genotype. We hypothesized that all patients would make some degree of motor improvement post-therapy, although those who possessed the BDNF-66Met or APOE-ε4 alleles would have less improvement with a standardized dose of therapy.

## Materials and Methods

### Participants

Genetic samples were obtained from 55 community-dwelling stroke patients aged 18–83 years (61.4 ± 13.8 years, mean ± SD) and 2–123 months poststroke (21.0 ± 3.1 months, mean ± SEM). All had suffered an unilateral stroke resulting in hemiparesis involving an upper limb. The present study was conducted as an optional adjunct to ongoing therapy trials in our laboratory. Inclusion criteria included (i) ≥14 years of age, (ii) medically stable, and (iii) cognitively competent as assessed by a Mini-Mental State Examination score ≥24. Exclusion criteria included (i) enrollment in any other formal rehabilitation program during the trial, (ii) comorbidities significantly affecting upper-limb sensorimotor function (e.g., diabetic neuropathy), and (iii) known infection with the blood-borne viruses HIV and/or hepatitis. All participants gave informed, written consent, and the study was approved by the Human Research Ethics Committee of St Vincent’s Hospital Sydney and conducted in accordance with the Declaration of Helsinki.

### Genotyping

A single venous blood sample (9 mL) was collected from each patient either pre- or post-therapy. Genomic DNA was isolated from leukocytes using the Autopure LS nucleic acid purification system (QIAgen, Hilden, Germany) at Genetic Repositories Australia, Sydney. DNA isolates were then amplified and used in separate assays to genotype each sample for the BDNF-Val66Met polymorphism and APOE-ε4 allele, as described below.

#### BDNF Genotyping

Isolated DNA was amplified using polymerase chain reactions (PCR) with the forward and reverse primers 5′-TGTATTCCTCCAGCAGAAAGAGAA-3′ and 5′-AAAGAAGCAAACATCCGAGGAC-3′, respectively. Reactions were performed using 40 ng of genomic DNA in a 25-μL total volume containing 50 mM MgCl_2_, 2.5 mM deoxynucleotide triphosphates, 10 pmol of each of the primers, and Platinum Taq polymerase (Life Technologies, Australia). The amplified 277 bp DNA fragment was digested using the restriction enzyme *Afl*III to give a 71-bp digestion control band in addition to a 206-bp band for the Met allele or 129 and 77 bp bands for the Val allele. PCR products were separated on 4% agarose gels stained with RedSafe (Intron Biotechnology, South Korea) and viewed using a UV transilluminator and Image Lab software (Bio-Rad Laboratories, Australia). Genotypes were read by two blinded assessors and the results corroborated to ensure reliability. In addition, a blind repeat of the BDNF assay was conducted on all samples. No discrepancies were identified for any sample between the two blinded assessors or between the repeat assays.

#### APOE Genotyping

TaqMan SNP genotyping assays, C_3084793_20 and C_904973_10 (AB Applied Biosystems, Life Technologies, Australia), were conducted on genomic DNA to genotype the two APOE-SNPs rs429358 and rs7412, respectively. The composition of these two SNPs was used to determine APOE genotype, as outlined in Table 1. Allelic discrimination assays were performed in 5 μL total volume in 384-well plates, using SNP specific primers and probes on an ABI 7900HT Fast Real-Time PCR instrument according to manufacturer’s instructions (AB Applied Biosystems, Life Technologies, Australia). Blind repeats of both assays were completed to ensure reliability of the results. As for BDNF, all APOE genotypes were read by two independent assessors with 100% inter-rater and inter-assay concordance.

**Table 1:** **APOE genotype, based on allelic distribution at two SNP loci**.

rs429358		rs7412		APOE genotype	*n* (%)
Nucleotides	Amino acid 112	Nucleotides	Amino acid 158		
T/T	Cys/Cys	T/T	Cys/Cys	ε2/ε2	1 (1.8)
T/T	Cys/Cys	C/T	Arg/Cys	ε2/ε3	8 (14.8)
T/T	Cys/Cys	C/C	Arg/Arg	ε3/ε3	36 (66.7)
T/C	Cys/Arg	C/T	Arg/Cys	ε2/ε4	2 (3.7)
T/C	Cys/Arg	C/C	Arg/Arg	ε3/ε4	7 (12.9)
C/C	Arg/Arg	C/C	Arg/Arg	ε4/ε4	0 (0)

### Motor-Function Assessments

A suite of functional assessments was used to quantify motor ability of the more-affected upper limb. This included the timed tasks of the Wolf Motor Function Test (WMFT-tt) (48), upper-limb motor Fugl-Meyer Assessment (FMA) (49), and Motor Activity Log Quality of Movement scale (MALQOM) (50). These assessments were completed by all patients both immediately before (pre-therapy/baseline) and after (post-therapy) a 14-day protocol of upper-limb rehabilitation (details below).

**Figure 1:**
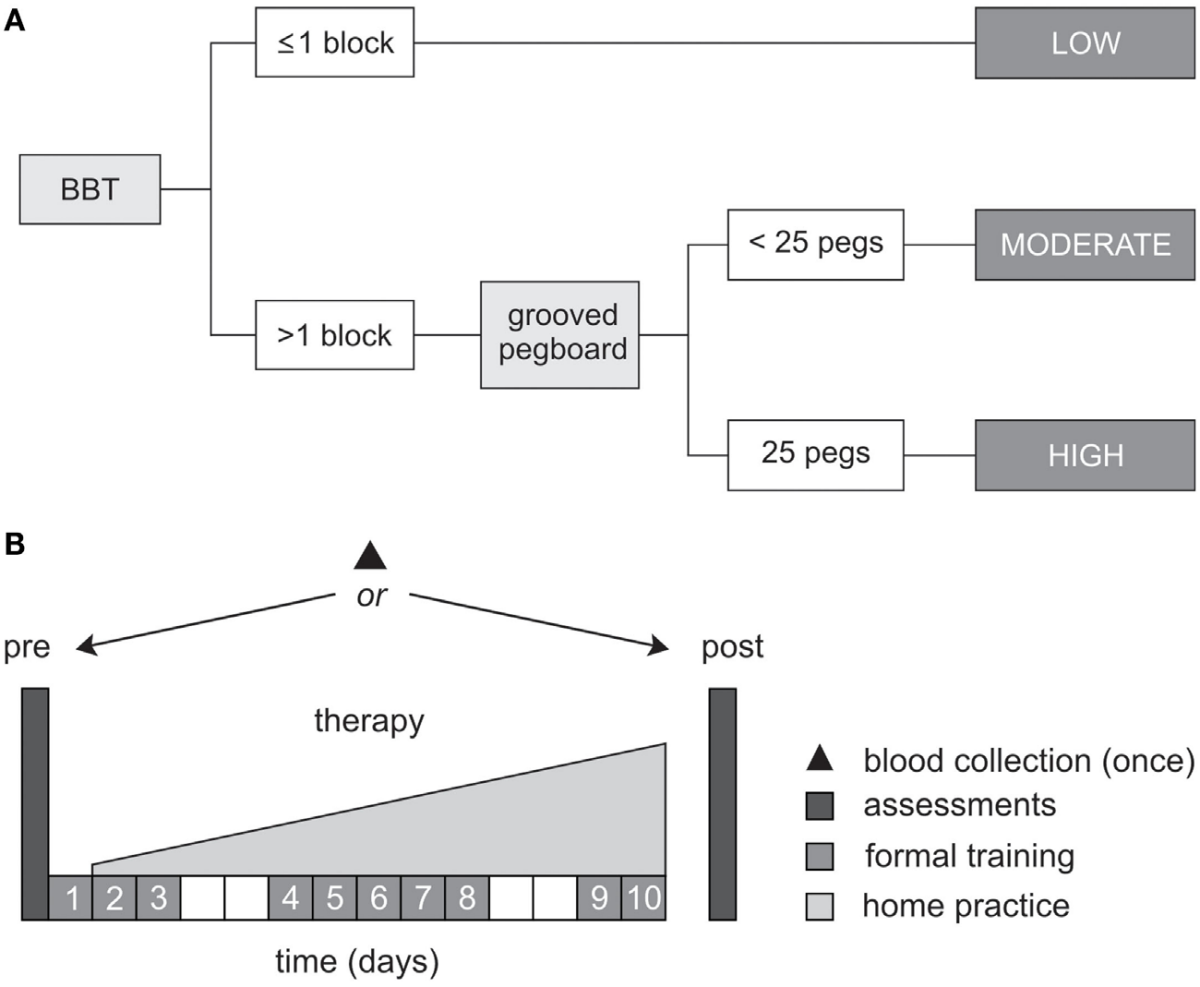
**Study protocol**. **(A)** Schematic representation of the objective classification scheme developed by Thompson-Butel and colleagues (51). The box and block test (BBT) and grooved pegboard were used prior to therapy to classify each patient with low, moderate, or high motor function. **(B)** All patients completed a dose-matched 14-day protocol of upper-limb rehabilitation, involving 10 formal therapy sessions and progressively increasing home practice (white squares represent weekends). Functional motor ability was assessed pre- and post-therapy, *via* the Wolf Motor Function Test timed tasks, the upper-limb motor Fugl-Meyer Assessment, and the Motor Activity Log Quality of Movement Scale. A single blood sample was collected from each patient for BDNF and APOE genotyping.

During pre-therapy assessments, two tests of dexterity were used to classify the level of upper-limb motor function, according to the objective classification scheme developed by Thompson-Butel and colleagues (51). In brief, patients unable to pick up and move >1 block on the box and block test (BBT) were classified with low motor function, while those who could move >1 block progressed to the more challenging grooved pegboard test. Patients unable to complete the grooved pegboard were classified with moderate motor function while those who successfully placed all 25 pegs were classified with high motor function (see Figure 1A).

### Upper-Limb Therapy

All patients completed a dose-matched 14-day protocol of either Wii-based Movement Therapy (*n* = 40) or modified Constraint-induced Movement Therapy (*n* = 15), as part of ongoing rehabilitation trials within the authors’ laboratory group. Therapy allocation was determined by the rehabilitation trial each patient was enrolled in and was not influenced by this adjunct genetic study. Of the present cohort, 28 patients were randomly allocated to either Wii-based Movement Therapy or modified Constraint-induced Movement Therapy (13 and 15 patients, respectively), as part of a randomized-controlled trial comparing these two-therapy protocols (47). The remaining 27 patients were enrolled in trials specifically investigating Wii-based Movement Therapy. Therapy outcomes for patients in the present study appear in the following publications: McNulty and colleagues (47) (*n* = 29), Shiner and colleagues (52) (*n* = 4), Thompson-Butel and colleagues (53) (*n* = 3), and *n* = 19 remain unpublished.

Between-therapy differences were not anticipated in the present study because the two therapies are founded on the same core principles, were identically dose matched, and random patient allocation was performed (47). For both therapies, 10 formal therapy sessions of 60-min duration were administered by an Accredited Exercise Physiologist on consecutive weekdays, specifically targeting movement of the more-affected hand and arm. Formal therapy was augmented by progressively increasing home practice which began on day 2 of the protocol and continued until day 14 (see Figure 1B).

Wii-based Movement Therapy uses the Nintendo Wii and Wii Sports program (Nintendo, Japan) under therapist guidance as a structured upper-limb rehabilitation tool (47, 54, 55). Patients operate the controller using only the more-affected hand and interact with the games of Wii-golf, -bowling, -baseball, -boxing, and -tennis using targeted movements of the more-affected arm. Modified Constraint-induced Movement Therapy promotes forced use of the more-affected hand and arm by constraining the less-affected hand in a padded mitt for up to 90% of waking hours. Constraint therapy and home-practice activities involve part-task training based on object manipulation with a focus on movement speed (56, 57).

### Data Analysis

Assay data were used to classify each patient as either a carrier or non-carrier of the BDNF-66Met and APOE-ε4 alleles, pooling homozygotes and heterozygotes for each polymorphism (16). Since there were no differences in either baseline motor function or therapy improvements between patients who completed Wii-based Movement Therapy and modified Constraint-induced Movement Therapy, all analyses were conducted on the combined patient cohort. Functional assessment data were not normally distributed and could not be transformed to a normal distribution; therefore, non-parametric statistical methods were used for analyses. At baseline, Mann–Whitney *U* tests were used to assess differences in age and pre-therapy motor function between carriers and non-carriers of each allele, while Chi-squared analyses were used to assess between-group differences in categorical variables including sex, ethnicity, and stroke etiology. Wilcoxon Signed-Rank tests were used to examine changes in functional movement ability induced by therapy. Generalized linear mixed models were then used to investigate the influence of genotype and motor function on improvements with therapy assessed using the WMFT-tt and FMA. Separate models were implemented for each gene (BDNF, APOE), where the dependent variable was post-therapy score, controlling for pre-therapy score and with fixed factors of genotype (carrier, non-carrier), and motor functional classification (low, moderate, and high). A fixed interaction between genotype and motor functional classification was also entered into the model for BDNF, but the sample size precluded a similar analysis for APOE (see Results). All statistical analyses were conducted on raw data using SPSS 21 software (IBM, USA), and differences were considered significant when *p* < 0.05. Parametric data are reported as mean ± SEM, while non-parametric data are reported as median (interquartile range) in the text, and presented as mean ± SEM in figures for greater clarity.

## Results

### Genotype

Brain-derived neurotrophic factor and APOE genotypes could be determined for 54/55 patients, due to insufficient DNA yield and quality for one patient sample. Twenty-seven patients were identified as Val/Val homozygotes, 24 as Val/Met heterozygotes, and 3 as Met/Met homozygotes. Thus, 27 patients possessed at least one copy of the BDNF-66Met allele (“Met carriers”), while 27 did not (“non-carriers”). For APOE, 9 patients were identified as ε4 carriers, and 45 as non-ε4 carriers (see Table 2 for full breakdown). Despite being sampled from a multi-ethnic population, both BDNF and APOE genotype frequencies were in Hardy–Weinberg equilibrium (BDNF: *χ*^2^ = 0.25, *p* = 0.617, APOE: *χ*^*2*^ = 0.562, *p* = 0.453) and conformed to expected population rates (20, 40).

**Table 2:** **Demographic characteristics and baseline motor function, according to BDNF-Val66Met and APOE-ε4 status**.

	BDNF Val66Met			*APOE* ε*4*			All
	*Met* carriers	Non-Met-carriers	*p* Value	ε4 carriers	Non ε4 carriers	*p* Value	
*n* (%)	27 (50)	27 (50)	–	9 (16.7)	45 (81.8)	–	54 (100)
Age	59.1 ± 16.8	63.6 ± 10.0	0.28	62.4 ± 9.6	61.2 ± 14.6	0.84	61.4 ± 13.8
Sex (F:M)	6:21	7:20	0.82	2:7	11:34	0.77	13:41
Ethnicity (Caucasian:Asian:Hispanic)	20:7:0	25:1:1	0.20	8:1:0	37:7:1	0.73	45:8:1
Dominant side (right:left)	24:3	26:1	0.30	8:1	42:3	0.64	50:4
More-affected side (right:left)	14:13	13:14	0.16	6:3	21:24	0.08	27:27
Stroke type (isch:haem)	18:9	20:7	0.55	7:2	31:14	0.59	38:16
Months poststroke	29.4 ± 4.5	28.8 ± 5.5	0.82	22.4 ± 4.8	30.5 ± 4.1	0.77	21.0 ± 3.1
Motor function, *n*							
Low	9	10	0.81	1	18	0.09	19 (35.2)
Moderate	8	9		5	12		17 (31.5)
High	10	8		3	15		18 (33.3)
Pre-therapy WMFT-tt (s)	38.7 ± 7.3	42.3 ± 7.9	0.46	24.6 ± 10.0	43.4 ± 6.0	0.14	40.0 ± 5.3
Pre-therapy FMA	46.1 ± 3.6	42.0 ± 3.9	0.36	50.0 ± 5.4	42.8 ± 2.9	0.35	44.1 ± 2.6

*Age is reported as mean ± SD, remaining data are reported as mean ± SEM*.

*Isch, ischemic; haem, haemorrhagic; WMFT-tt, mean time for the Wolf Motor Function Test timed tasks (maximum time of 120 s where lower time indicates better motor function); FMA, upper-limb Fugl-Meyer Assessment (maximum score 66)*.

*Motor function classified according to the scheme of Thompson-Butel and colleagues (51)*.

### Baseline Motor Function

Demographic characteristics and baseline motor function for carrier/non-carrier groups are reported in Table 2. A single subject was <3 months poststroke, and stability of pre-therapy motor function was confirmed by repeat pre-baseline and baseline motor function assessments 14 days apart. The remainder of the cohort were all >3 months poststroke, with an average of 21.0 ± 3.1 months poststroke. The pooled cohort was heterogeneous for upper-limb motor function, whereby baseline WMFT-tt mean scores ranged from 1.9 to 113.1 s (maximum time is 120 s). This heterogeneity was well balanced across BDNF/APOE carriers and non-carriers, with no significant differences between groups for any demographic variable or any measure of motor function at baseline, for either BDNF or APOE (see Table 2). Similarly, as in previous work (47), there were no significant differences in motor function at baseline according to therapy allocation (Wii-based Movement Therapy vs. modified Constraint-induced Movement Therapy: WMFT-tt, *p* = 0.83; FMA, *p* = 0.76; MALQOM, *p* = 0.73).

### Motor Improvements with Therapy

**Figure 2:**
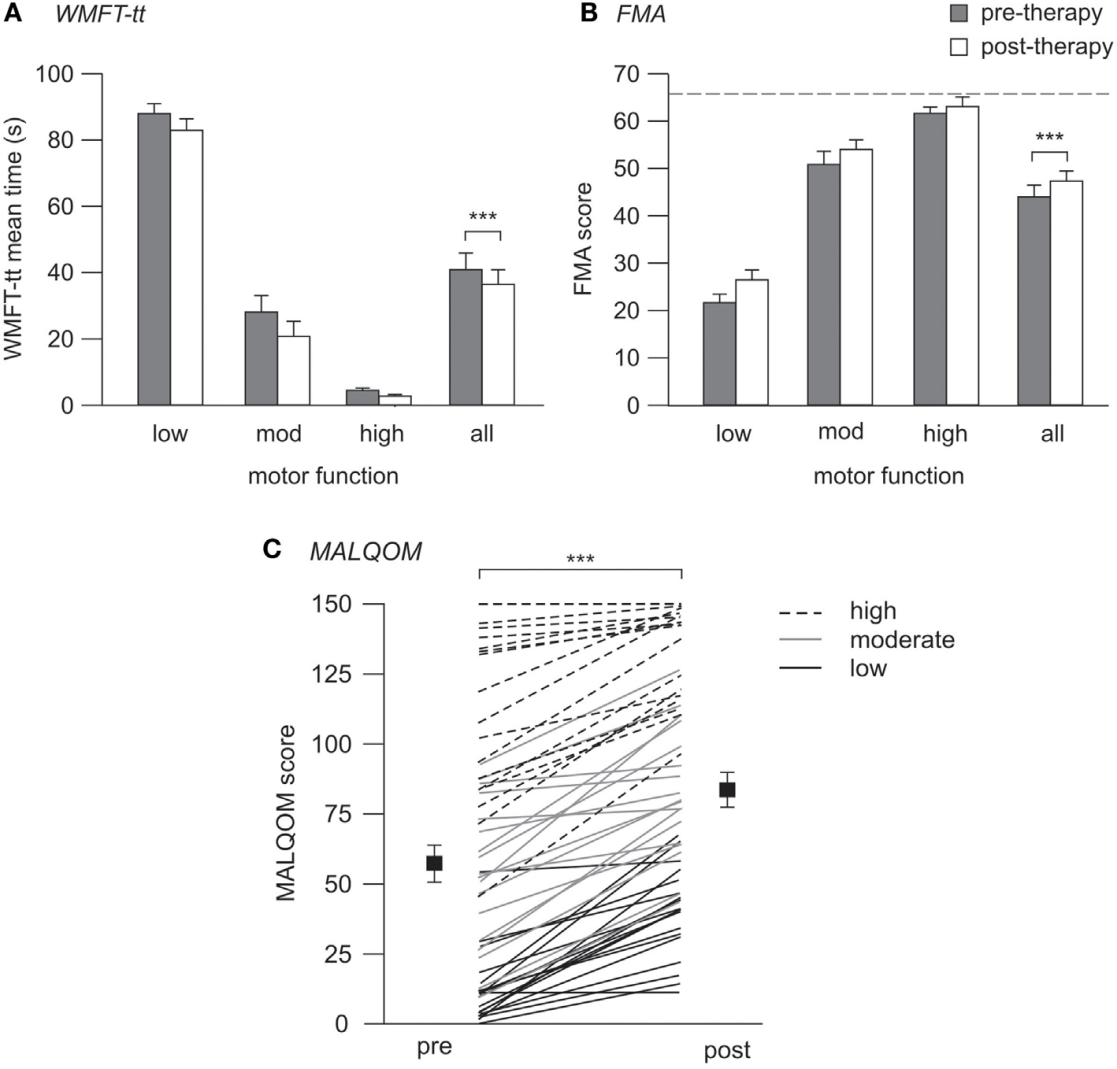
**Pre- and post-therapy motor function**. **(A,B)** contain mean data and SEs, **(C)** contains individual patient data (lines) with mean (squares) and SEs. **(A)** WMFT-tt mean times pre- (gray) and post-therapy (white), where lower time indicates better motor function. **(B)** FMA scores pre- and post-therapy, where higher score indicates better motor function, and dotted line indicates the maximum test score of 66. **(C)** Pre- and post-therapy MALQOM scores for all patients illustrating the wide range of motor function within the cohort, spanning the entire test range of 0–150. Significant improvement pre- to post-therapy was evident on all three measures (***p < 0.001).

Motor function significantly improved across the cohort post-therapy on all measures tested. Median time for the WMFT-tt reduced from 22.3 s (4.0–81.6) to 20.4 s (2.8–74.4) (*p* < 0.001), indicating faster and more efficient movement post-therapy. Similarly, upper-limb FMA scores increased from 48.0 (26.3–62.0) to 54.0 (30.3–62.8) pre- to post-therapy (*p* < 0.001). These improvements were evident in all patients and translated to improved performance of activities of daily living using the more-affected upper limb, as quantified using the MALQOM (57.7 ± 6.4–84.1 ± 5.8 pre- to post-therapy, *p* < 0.001) (see Figure 2). There were no differences between Wii-based Movement Therapy and modified Constraint-induced Movement Therapy patients for any measure of therapy-induced improvement (WMFT-tt, *p* = 0.61; FMA, *p* = 0.47; MALQOM, *p* = 0.41).

### Influence of Genotype and Motor Function on Improvements with Therapy

#### Brain-Derived Neurotrophic Factor

**Figure 3:**
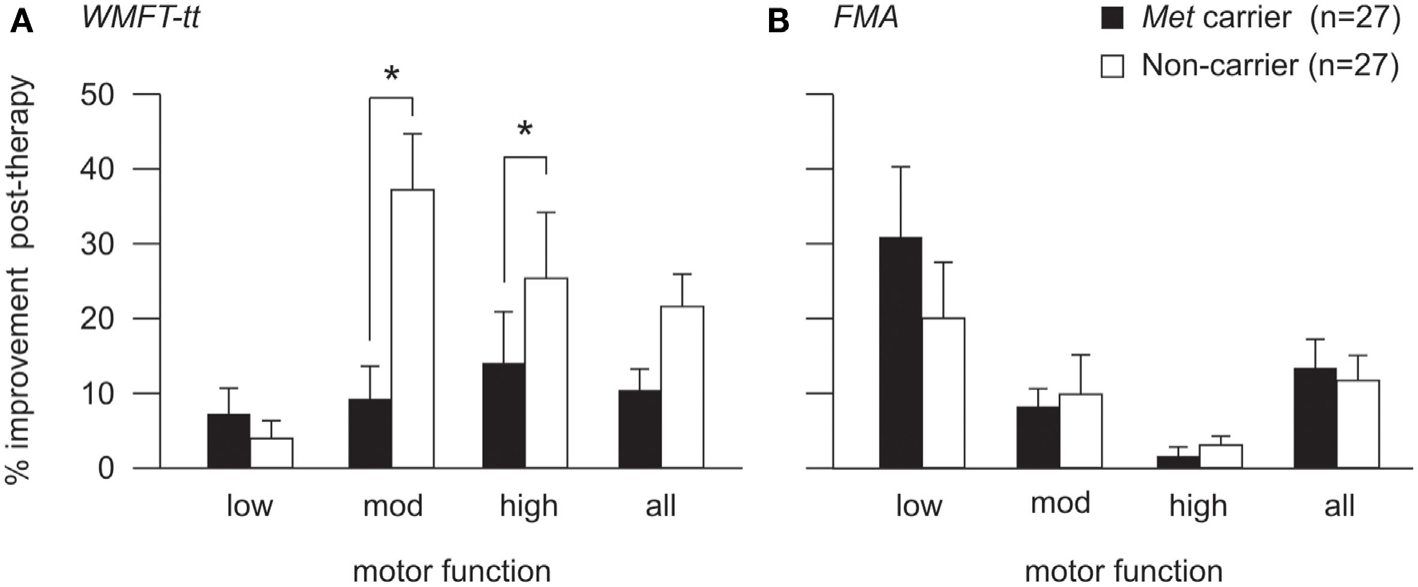
**Post-therapy improvement according to BDNF genotype and motor function**. Post-therapy improvement on the WMFT-tt **(A)** and FMA **(B)** for BDNF Met carriers (black) and non-Met carriers (white) with high, moderate, and low motor function. Improvement is illustrated as percentage change from pre-therapy score (mean and SE). There was a significant interaction between BDNF genotype and motor function for the WMFT-tt (*p* = 0.029), whereby the Met allele was associated with lower proportional improvement for patients with high (*p* = 0.046) and moderate (*p* = 0.008), but not low motor function **(A)**. The same pattern was observed for FMA data **(B)**, which was more sensitive to improvements made by patients with low motor function, although this did not reach statistical significance (*p < 0.05). For detailed breakdown of the number of carriers/non-carriers with each level of motor function, please refer to Table 2.

For the WMFT-tt data, generalized linear mixed modeling identified a significant fixed effect of baseline motor function on therapy-induced improvement [*F*(2,47) = 3.32, *p* = 0.045], whereby patients with high and moderate motor function made greater improvements than those with low motor function on this assessment. No significant main effect of BDNF genotype was identified for the cohort as a whole [*F*(1,47) = 0.439, *p* = 0.15]. However, a significant interaction between BDNF genotype and motor function was identified [*F*(2,47) = 3.81, *p* = 0.029], whereby the BDNF-Met allele had an effect on therapy-induced improvements for patients with high (*p* = 0.046) and moderate (*p* = 0.008), but not low motor function (see Figure 3A). Met carriers with high and moderate motor function made proportionally less improvement than respective non-carriers (high: 13.8 ± 6.8 vs. 25.2 ± 8.9%; moderate: 8.9 ± 4.7 vs. 37.3 ± 7.5%), while Met carriers/non-carriers with low motor function made comparable post-therapy gains (7.0 ± 3.7 vs. 3.8 ± 2.4%, respectively).

To ensure that this genotype by motor-function interaction was not driven by an artifact of test sensitivity, generalized linear mixed modeling was also performed using FMA data, which is a more salient measure of motor improvement for patients with low motor function than the WMFT-tt (58). The interaction between BDNF genotype and motor function did not reach statistical significance in the FMA model [*F*(2,47) = 2.071, *p* = 0.137] but the same pattern was observed, whereby the presence of the Met allele appeared to associate with less proportional improvement post-therapy for patients with high and moderate, but not low motor function (Figure 3B).

#### Apolipoprotein E

Given the demographic breakdown of ε4 carriers (*n* = 9 in total, with only one patient with low motor function, see Table 2), our sample size was not sufficient to examine the interaction between APOE genotype and motor function in the mixed model. Instead, only the main fixed effects of motor function and APOE genotype for pooled ε4 carriers vs. non-carriers were investigated. There was a trend for a fixed effect of motor-functional classification on post-therapy improvements when quantified using the WMFT-tt [*F*(2,50) = 3.03, *p* = 0.057]. However, no significant effect of APOE genotype on post-therapy improvement was evident when quantified using either the MALQOM [*F*(1,50) = 0.26, *p* = 0.61] or the FMA [*F*(1,50) = 0.45, *p* = 0.51].

## Discussion

This study explored the relationships between BDNF and APOE genotype, chronic motor status, and motor improvement following a 14-day protocol of upper-limb stroke therapy. In a heterogeneous, community-dwelling stroke cohort, we saw no effect of either the BDNF-66Met or the APOE-ε4 allele on patients’ level of residual voluntary motor function at baseline. Similarly, BDNF and APOE genotype did not significantly influence the magnitude of post-therapy improvements for the pooled cohort data. However, when patients were classified according to their baseline level of residual voluntary motor function, we identified a novel interaction between BDNF genotype and therapy improvements, whereby BDNF-Met carriers achieved less improvement relative to non-carriers among those patients with high and moderate, but not low motor function (Figure 3). This is the first demonstration that the BDNF-Val66Met polymorphism may have a differential effect on rehabilitation responsiveness depending on baseline motor function. These data suggest that different mechanisms of recovery mediate improvements for patients with different levels of residual motor function and illustrate that motor-function status should be considered together with BDNF genotype during the design and stratification of clinical trials.

### An Interaction between BDNF Genotype and Poststroke Motor Function

Distinctly different improvement patterns for BDNF-Met carriers vs. non-carriers became apparent when examining the data according to patients’ level of residual motor function. Met carriers with high and moderate motor function improved by 50 and 25%, respectively, compared to non-carriers, while the Val66Met polymorphism had no significant effect for patients with low motor function (see Figure 3). There was even some suggestion of greater average improvement for Met carriers relative to non-carriers in the low motor function subgroup, although this reversed pattern did not reach statistical significance. The interaction between BDNF genotype and motor status suggests the improvement for patients with different levels of motor ability may be mediated by different mechanisms. We hypothesize that patients with high and moderate motor function may rely primarily on cortical plasticity processes that are directly affected by the Val66Met polymorphism, while those with low motor function and possibly more extensive cortical damage, rely more on subcortical plasticity processes that are less influenced by BDNF (27). Improvement in patients with low residual motor function may also relate to the extent of primary damage and the integrity of remaining cortical structures available to generate motor output (59), making a more subtle effect of genotype difficult to discern in this cohort.

In acute stroke, Di Lazzaro and colleagues recently demonstrated that the BDNF-Val66Met polymorphism can have a differential effect on intracortial excitability parameters (60). Non-carriers of the polymorphism were seen to have greater excitability changes poststroke, with more pronounced hemispheric differences in the balance of excitability. It is possible that the functional relevance of such excitability changes may vary according to the amount of damage, and hence residual impairment poststroke (61, 62). This may be one mechanism *via* which BDNF genotype may interact with baseline motor function to influence motor outcomes, although further investigations that incorporate genetic, motor, and cortical excitability testing in the chronic period are necessary.

An interaction between BDNF genotype and baseline motor function may help explain the lack of consensus in the literature regarding the role of the Val66Met polymorphism in stroke recovery (27). In the present study, a significant effect of Val66Met across the pooled cohort was only apparent after stratifying the cohort according to motor function. In previous negative trials [e.g., Ref. (16, 33, 34)], significant gene effects may have been masked by considering only pooled data from a cohort with varied motor function. Thus, stratification according to motor-function status may be necessary to elucidate gene effects in heterogeneous cohorts in chronic stroke. Our data also provide further evidence for the clinical utility of the stratification scheme developed by Thompson-Butel and colleagues (51).

### Implications for Therapy Prescription, Dosage, and Trial Design

An important finding from this study is that while the Val66Met polymorphism has the capacity to influence the relative magnitude of improvement following a standard dose of therapy, neither the BDNF-66Met nor the APOE-ε4 allele *precludes* improvement. Post-therapy improvements were evident in all patients including carriers of both the Met and ε4 alleles and across all levels of baseline motor function (Figures 2 and 3). While improvements may be more modest for Met carriers compared to non-carriers, it should *not* be assumed that Met carriers do not have the capacity for improvement. In the present study, we quantified improvements after a standardized dose of therapy and so cannot be certain whether Met carriers would have achieved similar gains to non-carriers given additional or more intensive therapy. There is some suggestion that intensive training in a healthy population can overcome the effect of the Val66Met polymorphism with regard to short-term plasticity (63), and thus it is possible that a higher dose or more intensive therapy may also lessen the impact of the polymorphism in poststroke populations.

Our results suggest that BDNF genotype and baseline motor function together have important implications for therapy prescription and clinical trial design. We propose that this information should inform *how* therapy programs are structured and what therapy outcomes can be expected and not categorize *who* should or should not receive rehabilitation. Our data suggest that future studies should investigate if Met carriers with high and moderate motor function require more intensive therapy or longer therapy duration than non-carriers to achieve a comparable magnitude of improvement. Patients with low motor function made significantly less improvement on the WMFT-tt compared to those with higher baseline function, suggesting they may require a larger therapy dose and/or intensity regardless of BDNF genotype. However, FMA and MALQOM data confirm that substantial improvements were also evident in patients with low motor function when assessed using tools more appropriate for their motor-function status (Figures 2 and 3B). The observed effect of BDNF genotype and residual motor function on the magnitude of post-therapy improvement suggests that stratification may be advisable in future clinical trials investigating rehabilitation efficacy to ensure balance between trial arms. This would enable the inclusion of Met carriers and/or patients with low motor function without introducing inadvertent study bias and would so improve the generalizability of results.

On initial inspection, post-therapy WMFT-tt and FMA data may appear incongruous, whereby patients with low motor function appear to make greater improvements according to the FMA, while patients with high and moderate motor function appear to improve more on the WMFT-tt (see Figure 3). We interpret this as a reflection of the sensitivity of these two assessment tools for patients with different levels of motor function. It is well established that the WMFT-tt can have a prominent floor effect for patients with low motor function, while the FMA can have limited sensitivity and a ceiling effect for those with high motor function (58, 64–66). Thus, both these assessments have differing sensitivity across the spectrum of poststroke motor impairment, emphasizing that a suite of complementary assessment tools is more informative than any single assessment (58). Our MALQOM data support this interpretation by demonstrating prominent therapy-induced improvements for patients across the entire cohort, from low to high motor function (see Figure 2C). Moreover, the same pattern of interaction between BDNF genotype and motor function was evident for both WMFT-tt and FMA data, regardless of apparent differences in improvement magnitude (see Figure 3). It is likely that limited FMA sensitivity for patients with high and moderate motor function contributed to this genotype by motor-function interaction not reaching statistical significance for FMA data.

### Study Limitations

While considerable in size for a chronic intervention study, we acknowledge this is a small sample for genetic analyses. We, therefore, adopted a candidate gene rather than genome-wide approach and focused on two polymorphisms that are the best characterized and prevalent in the population. Our sample included sufficient BDNF-Met carriers to enable the investigation of an interaction between BDNF genotype and motor function. A lower proportion of ε4 carriers precluded this analysis for APOE and likely contributed to our lack of significant APOE findings. Statistical power limited our investigation to the comparison of “carriers” vs. “non-carriers,” and thus, we cannot comment on any possible gene dosage effects for heterozygotes compared to homozygotes. Similarly, we were not able to investigate possible gene–gene interactions, which may be of interest (67, 68).

Stroke is a heterogeneous disease in many ways, and here, we focus on clinical motor heterogeneity. A much larger data set is required to consider the influence of additional covariates, such as age (69), sex (70, 71), stroke etiology (72, 73), and time poststroke, that have the capacity to interact and therefore introduce complex variability. We acknowledge that inclusion of two different therapy modalities may have introduced subtle variability, where a lack of significant difference between the groups may not necessarily indicate statistical equivalence. However, use of a combined cohort provided superior statistical power for our primary genetic analyses of interest, as in a previous study (16).

## Conclusion

This study identified a novel and significant interaction between BDNF-Val66Met genotype and motor function on the magnitude of improvement following a protocol of post-acute stroke rehabilitation. Here, the BDNF-Met allele was associated with less proportional improvement following a standard dose of therapy for patients with high and moderate, but not low motor function. These data suggest that different mechanisms of recovery may be important for patients of different levels of residual voluntary motor capacity, and stratification according to motor function may be necessary to elucidate gene effects in heterogeneous stroke cohorts. Neither the BDNF-66Met nor the APOE-ε4 allele precluded improvement in carriers, emphasizing that given appropriate rehabilitation both carriers and non-carriers retain the capacity for improvement. Thus, we propose genetic information should be used to guide optimal therapy prescription and enable stratification in clinical trials, rather than to determine therapy allocation.

## Author Contributions

CS collected and analyzed data and drafted and approved the manuscript. KP coanalyzed genetics data and approved the manuscript. AT-B completed stroke therapy and approved the manuscript. TT cocollected data and approved the manuscript. PS designed genetic analysis and approved the manuscript. PMcN conceived and supervised the study and revised and approved the manuscript.

## Conflict of Interest Statement

The authors declare that the research was conducted in the absence of any commercial or financial relationships that could be construed as a potential conflict of interest.
